# Correction: Does papillary muscle free strain has predictive value in risk stratification of patients with hypertrophic cardiomyopathy?

**DOI:** 10.1371/journal.pone.0314401

**Published:** 2024-11-20

**Authors:** Atilla Koyuncu, Cennet Yildiz, Lutfi Ocal, Sedat Kalkan, Alev Kılıçgedik, Mustafa Ozan Gürsoy, Ersan Oflar, Gökhan Kahveci

The third author’s name is spelled incorrectly. The correct name is: Lutfi Ocal. The correct citation is: Koyuncu A, Yildiz C, Ocal L, Kalkan S, Kılıçgedik A, Gürsoy MO, et al. (2023) Does papillary muscle free strain has predictive value in risk stratification of patients with hypertrophic cardiomyopathy? PLOS ONE 18(2): e0282054. https://doi.org/10.1371/journal.pone.0282054.
