# Correction: Does papillary muscle free strain has predictive value in risk stratification of patients with hypertrophic cardiomyopathy?

**DOI:** 10.1371/journal.pone.0341793

**Published:** 2026-01-28

**Authors:** The PLOS One Editors

## Notice of Republication

This article [1,2] was republished on January 7, 2026, to address an issue identified post-publication. Please download this article again to view the correct version.

The article’s Data Availability statement is updated to: The data used in this study cannot be shared publicly as they contain potentially identifying and sensitive patient information. Although the dataset has been de-identified, there remains a risk of re-identification due to the nature of the clinical variables collected. In addition, the data are owned by a third-party institution, Bakırköy Dr. Sadi Konuk Training and Research Hospital, and may only be shared in accordance with the hospital’s data protection policies and Turkish data privacy regulations. Ethical and legal restrictions on data sharing have been imposed by the Bakırköy Dr. Sadi Konuk Training and Research Hospital Ethics Committee, which approved the study protocol. Researchers who meet the criteria for access to confidential data (e.g., holding appropriate institutional ethics approval and signing a data use agreement) may request access to the dataset through the Ethics Committee at gulnur.yilmaz2@saglik.gov.tr.
