# Supplementary material for: Does papillary muscle free strain has predictive value in risk stratification of patients with hypertrophic cardiomyopathy?
Source: PLoS One. 2023 Feb 24;18(2):e0282054. doi: 10.1371/journal.pone.0282054 (PMC9955626; doi:10.1371/journal.pone.0282054)
Supplement: Attachment — Submitted filename: RESPONSE TO REVIEWERS.docx [file pone.0282054.s001.docx]

PONE-D-22-24350

Does papillary muscle free strain has predictive value in risk stratification of patients with hypertrophic cardiomyopathy?

PLOS ONE

Dear Dr. YILDIZ,

Thank you for submitting your manuscript to PLOS ONE. The MS was reviewed by two referees. One requested major revision, the other wanted the MS to be rejected. My impression is also major revision, but this revision must be done very thoroughly and address sufficiently all the points the reviewers raised.

In addition: My concern is the correlation strain vs. sudden cardiac death. Though mathematically correct (I assume) the data points show rather uncorrelated data in the low and mid risk group. Only the high risk group shows higher strains. However, these values are not much higher those in the upper range of the low and mid risk group. Or to put it the other way round: if you find a high strain, you do not know much about the risk of the patient. Only when the strain is appropriately low, the risk of sudden cardiac death is low. So strain has a more negative prognostic value.  Please comment on this. 


Please submit your revised manuscript by Nov 03 2022 11:59PM. If you will need more time than this to complete your revisions, please reply to this message or contact the journal office at [plosone@plos.org](mailto:plosone@plos.org). When you're ready to submit your revision, log on to https://www.editorialmanager.com/pone/ and select the 'Submissions Needing Revision' folder to locate your manuscript file.

Please include the following items when submitting your revised manuscript:

- A rebuttal letter that responds to each point raised by the academic editor and reviewer(s). You should upload this letter as a separate file labeled 'Response to Reviewers'.
- A marked-up copy of your manuscript that highlights changes made to the original version. You should upload this as a separate file labeled 'Revised Manuscript with Track Changes'.
- An unmarked version of your revised paper without tracked changes. You should upload this as a separate file labeled 'Manuscript'.

If you would like to make changes to your financial disclosure, please include your updated statement in your cover letter. Guidelines for resubmitting your figure files are available below the reviewer comments at the end of this letter.

If applicable, we recommend that you deposit your laboratory protocols in protocols.io to enhance the reproducibility of your results. Protocols.io assigns your protocol its own identifier (DOI) so that it can be cited independently in the future. For instructions see: <https://journals.plos.org/plosone/s/submission-guidelines#loc-laboratory-protocols>. Additionally, PLOS ONE offers an option for publishing peer-reviewed Lab Protocol articles, which describe protocols hosted on protocols.io. Read more information on sharing protocols at <https://plos.org/protocols?utm_medium=editorial-email&utm_source=authorletters&utm_campaign=protocols>.

We look forward to receiving your revised manuscript.

Kind regards,

Wolfgang Rudolf Bauer, M.D., Ph.D.
Academic Editor
PLOS ONE

Journal Requirements:

When submitting your revision, we need you to address these additional requirements.

1. Please ensure that your manuscript meets PLOS ONE's style requirements, including those for file naming. The PLOS ONE style templates can be found at 
<https://journals.plos.org/plosone/s/file?id=wjVg/PLOSOne_formatting_sample_main_body.pdf> and 
<https://journals.plos.org/plosone/s/file?id=ba62/PLOSOne_formatting_sample_title_authors_affiliations.pdf>

- Manuscript has been revised according to PLOS ONE’s style requirements.

2. Please amend your current ethics statement to address the following concerns:
a) Did participants provide their written or verbal informed consent to participate in this study?
b) If consent was verbal, please explain i) why written consent was not obtained, ii) how you documented participant consent, and iii) whether the ethics committees/IRB approved this consent procedure.

- All the participants provided their written informed consent to participate in this study.

3. Thank you for stating the following financial disclosure: 
 "The funders had no role in study design, data collection and analysis, decision to publish, or preparation of the manuscript."

At this time, please address the following queries:

a) Please clarify the sources of funding (financial or material support) for your study. List the grants or organizations that supported your study, including funding received from your institution. 
b) State what role the funders took in the study. If the funders had no role in your study, please state: “The funders had no role in study design, data collection and analysis, decision to publish, or preparation of the manuscript.”
c) If any authors received a salary from any of your funders, please state which authors and which funders.
d) If you did not receive any funding for this study, please state: “The authors received no specific funding for this work.”

Please include your amended statements within your cover letter; we will change the online submission form on your behalf.

- Financial disclosure has been stated in the cover letter.

4. In your Data Availability statement, you have not specified where the minimal data set underlying the results described in your manuscript can be found. PLOS defines a study's minimal data set as the underlying data used to reach the conclusions drawn in the manuscript and any additional data required to replicate the reported study findings in their entirety. All PLOS journals require that the minimal data set be made fully available. For more information about our data policy, please see http://journals.plos.org/plosone/s/data-availability.

"Upon re-submitting your revised manuscript, please upload your study’s minimal underlying data set as either Supporting Information files or to a stable, public repository and include the relevant URLs, DOIs, or accession numbers within your revised cover letter. For a list of acceptable repositories, please see http://journals.plos.org/plosone/s/data-availability#loc-recommended-repositories. Any potentially identifying patient information must be fully anonymized.

Important: If there are ethical or legal restrictions to sharing your data publicly, please explain these restrictions in detail. Please see our guidelines for more information on what we consider unacceptable restrictions to publicly sharing data: http://journals.plos.org/plosone/s/data-availability#loc-unacceptable-data-access-restrictions. Note that it is not acceptable for the authors to be the sole named individuals responsible for ensuring data access.

We will update your Data Availability statement to reflect the information you provide in your cover letter.

- Minimal data set of the study have been available as Supporting Information files.

5. Please include your full ethics statement in the ‘Methods’ section of your manuscript file. In your statement, please include the full name of the IRB or ethics committee who approved or waived your study, as well as whether or not you obtained informed written or verbal consent. If consent was waived for your study, please include this information in your statement as well.

- Full name of the ethic committee has been provided in the Methods section. All patients gave written informed consent before study enrollment.

[Note: HTML markup is below. Please do not edit.]

Reviewers' comments:

Reviewer's Responses to Questions

**Comments to the Author**

1. Is the manuscript technically sound, and do the data support the conclusions?

The manuscript must describe a technically sound piece of scientific research with data that supports the conclusions. Experiments must have been conducted rigorously, with appropriate controls, replication, and sample sizes. The conclusions must be drawn appropriately based on the data presented.

Reviewer #1: Partly

Reviewer #2: No

2. Has the statistical analysis been performed appropriately and rigorously?

Reviewer #1: No

Reviewer #2: No

3. Have the authors made all data underlying the findings in their manuscript fully available?

The [PLOS Data policy](http://www.plosone.org/static/policies.action#sharing) requires authors to make all data underlying the findings described in their manuscript fully available without restriction, with rare exception (please refer to the Data Availability Statement in the manuscript PDF file). The data should be provided as part of the manuscript or its supporting information, or deposited to a public repository. For example, in addition to summary statistics, the data points behind means, medians and variance measures should be available. If there are restrictions on publicly sharing data—e.g. participant privacy or use of data from a third party—those must be specified.

Reviewer #1: No

Reviewer #2: Yes

4. Is the manuscript presented in an intelligible fashion and written in standard English?

PLOS ONE does not copyedit accepted manuscripts, so the language in submitted articles must be clear, correct, and unambiguous. Any typographical or grammatical errors should be corrected at revision, so please note any specific errors here.

Reviewer #1: Yes

Reviewer #2: Yes

5. Review Comments to the Author

Please use the space provided to explain your answers to the questions above. You may also include additional comments for the author, including concerns about dual publication, research ethics, or publication ethics. (Please upload your review as an attachment if it exceeds 20,000 characters)

Reviewer #1: In the present manuscript the authors try to answer the question whether papillary muscle free strain predicts the value in risk stratification of patients with hypertrophic cardiomyopathy? Beside the fact that it would be more important to analyze the potential influence on observed risk there some points that should be discussed.

They classified patients using the ESC risk calculator which tries to calculate the risk of sudden cardiac death (SCD) within the next 5 years. The model was developed in order to help clinicians in decision making of indication for ICD implantation in primary prevention.

The authors didn’t use the reported risk thresholds properly. High risk patients according ESC risk calculation model are defined as patients with a 5 years risk of SCD >6% - and NOT >4%. The model defines a risk >4 and <6% as patients of medium risk. Therefore, it is important to analyze the data using thresholds and definitions given in the guidelines. Furthermore, figure 2 should use another grading with steps of 2% calculated risk for SCD. The reported correlation is weak.

- We thank the reviewer. We recalculated the risk of the patients according to ESC risk calculation method (which includes seven variables) and divided into two groups according to their risks (low-intermediate risk group and high risk group). Our analysis showed that global longitudinal strain and anterior papillary muscle free strain were significantly reduced in patients with high risk scores. Correlation of sudden cardiac risk score with global longitudinal strain and anterolateral papillary muscle free strain have been depicted in Figure 2 and Figure 3, respectively. We found moderate correlation between sudden cardiac risk score and global longitudinal strain and anterolateral papillary muscle free strain.

It would be of clinical interest to give the values of the single risk factors which are used in the risk stratification model.

- We analyzed the single risk factors which were used in risk stratification model. According to our analysis patients who had high risk for SCD had higher LVOT gradient, higher rate of family history of SCD, non-sustained VT and syncope history.

Can the authors give information about patient follow-up including real risk of SCD.

- Unfortunately long-term follow-up of the patients was not done in the present study.

It does not make sense to give the percentages with 2 decimal places if only 55 patients were examined.

- We thank the reviewer. The percentages was given with one decimal places.

Did they measure LV gradients at rest or provocation?

- We thank the reviewer. LV gradients were measured at rest. It has been stated in the Methods section.

The chapter discussion includes some incorrect information like “Moreover almost one-quarter of HCMP patients had remaining high gradient after septal myectomy” or “However, in the past few decades histological abnormalities such as late gadolinium enhancement in cardiac MR studies or abnormalities in strain values have emerged as a new risk markers for adverse outcomes”

- We thank the reviewer. Incorrect information have been corrected. The sentence “Moreover almost one-quarter of HCMP patients had remaining high gradient after septal myectomy” has been removed from the discussion section.

Reviewer #2: Does papillary muscle free strain has predictive value in risk stratification of patients with hypertrophic cardiomyopathy?

Koyunzu et al. analyzed the transthoracic echocardiograms of 55 HCMP patients and 45 controls using 2D speckle-tracking imaging. The 55 HCMP patients were further subdivided according to their sudden cardiac death risk score (=4 points).
The authors found higher wall thickness and worse diastolic dysfunction in HCMP patients when compared to controls. They further found global longitudinal strain as well as papillary muscle strain values significantly different in HCMP patients. Global longitudinal strain and ALPM free strain were predictive of a high risk of SCD as calculated by the risk score with a low sensitivity but moderate to high specificity.
The manuscript deals with an iteresting topic. Nevertheless, there are several points to address:

Abstract:
• In the results part, a „-„ is missing before „11.97“.
• „ […]-15.88±12.73 vs -11.71±10.40, p=0.163, respectively“: either the p-value or the statement about significant group differences is not correct. Please revise.
• „(-16.70 (-19.37- -11.93) vs 11.97 (-18.57 - -7.90); p= 0.048 and -15.88±12.73 vs -11.71±10.40, p=0.163, respectively)“: it is unclear, which strain these values refer to? The global longitudinal strain or the two papillary muscle strains. Further, it is a bit confusing to present the one strain value as median and the other as mean. I would suggest to present both as median, if the mean cannot be presented due to skewed distribution.

- We thank the reviewer. All the numbers, p values have been revised and corrected. All the numbers in the abstract section were given as median and IQR.

Manuscript:

Page and line numbering would have been very helpful…

- We thank the reviewer. Page and line numbers have been added.

Material and Methods:

How did you identifiy the patients? Did you include consecutive patients presenting to your hospital or did you search the hospital clinical information system? How did you acquire informed consent?

- We thank the reviewer. In our hospital, echocardiographic examinations of the patients who had specific diseases were recorded and stored. Before echocardiographic examination, written informed consent of each patient was obtained. In the present study, we included consecutive patients who applied to our echocardiography department with the diagnosis of hypertrophic cardiomyopathy. Patients with poor image quality were excluded from the study.

How did you identify the controls?

- We thank the reviewer. After selection of HCMP patients, age and sex matched healthy controls were included in the study.

„SCD risk stratification of the patients were calculated according to the current guideline which includes five variables“ … there are 7 variables listed…

- We thank the reviewer. SCD risk stratification was done according to ESC guideline which includes seven variables. It has been corrected. SCD risk of each patient has been recalculated.

Were the echocardiograms performed according to a certain standard protocol? Who performed the echos?

- We thank the reviewer. Echocardiography was performed by a cardiologist who had experience in advanced echocardiography and trained for the requirements of the study. All of the echocardiographic examinations were done according to current guidelines.

Results:
„The mean age of the HOCM and the control group were […]“ you might have meant HCMP instead of HOCM?

- We thank the reviewer. It has been corrected.

„Intra-observer variability of two measures was found to be as 0.868 (CI 95%: 0.637-0.950).“ Please indicate, which parameter was analyzed. Usually, intra-observer variability varies for each parameter.

- We thank the reviewer. For evaluation of intra-observer variability, global longitudinal strain measurements were used. It has been stated in results section.

• Maximal LV wall thickness, LA diameter, and left ventricular outflow tract (LVOT) gradient, which all entered the risk score, have a strong relationship with LV strain values. A sensitivity analysis without these variables might be interesting to show an independent association of strain values and SCD risk.

- We thank the reviewer. In order to found the independent predictors of high SCD risk scores backward logistic regression analysis (with inclusion of parameters: Maximal LV wall thickness, LA diameter, and left ventricular outflow tract (LVOT) gradient) was conducted. The results showed that only age global longitudinal strain and ALPM free strain were the independent predictors of high SCD risk score.

Did you perform a follow-up to longitudinally assess the true risk of SCD/ICD shock?

- We thank the reviewer. Unfortunately long term follow-up of the patients were not done.

Was there an association of maximal LV wall thickness and papillary muscle strain?

- We thank the reviewer. Maximal LV wall thickness showed moderate correlation with anterolateral papillary muscle free strain (r=0.406, p=0.002). It has been stated in results section.

• Was an MRI available at least in some patients? Was there an association of LGE and papillary muscle strain?

- Unfortunately only small number of the patients (5 patients) had MRI studies. Since the number was so small, we could not make any statistical analysis.

In total, the incremental value of measuring papillary muscle strain, which is a sophisticated and time consuming method, does not become overt, so far.

- We thank the reviewer. According to our results ALPM free strain can be used to stratify HCMP patients for sudden cardiac risk. Risk stratification of HCMP patients could be challenging in some cases, we think that strain analysis could especially be useful in these group of subjects.

6. PLOS authors have the option to publish the peer review history of their article ([what does this mean?](https://journals.plos.org/plosone/s/editorial-and-peer-review-process#loc-peer-review-history)). If published, this will include your full peer review and any attached files.

If you choose “no”, your identity will remain anonymous but your review may still be made public.

**Do you want your identity to be public for this peer review?** For information about this choice, including consent withdrawal, please see our [Privacy Policy](https://www.plos.org/privacy-policy).

Reviewer #1: No

Reviewer #2: No

[NOTE: If reviewer comments were submitted as an attachment file, they will be attached to this email and accessible via the submission site. Please log into your account, locate the manuscript record, and check for the action link "View Attachments". If this link does not appear, there are no attachment files.]

While revising your submission, please upload your figure files to the Preflight Analysis and Conversion Engine (PACE) digital diagnostic tool, <https://pacev2.apexcovantage.com/>. PACE helps ensure that figures meet PLOS requirements. To use PACE, you must first register as a user. Registration is free. Then, login and navigate to the UPLOAD tab, where you will find detailed instructions on how to use the tool. If you encounter any issues or have any questions when using PACE, please email PLOS at [figures@plos.org](mailto:figures@plos.org). Please note that Supporting Information files do not need this step.

*In compliance with data protection regulations, you may request that we remove your personal registration details at any time.* [*(Remove my information/details)*](https://www.editorialmanager.com/pone/login.asp?a=r)*. Please contact the publication office if you have any questions.*
