# Supplementary material for: Does papillary muscle free strain has predictive value in risk stratification of patients with hypertrophic cardiomyopathy?
Source: PLoS One. 2023 Feb 24;18(2):e0282054. doi: 10.1371/journal.pone.0282054 (PMC9955626; doi:10.1371/journal.pone.0282054)
Supplement: Attachment — Submitted filename: RESPONSE TO REVIEWERS.docx [file pone.0282054.s002.docx]

We thank the reviewer for her/his careful reading of the manuscript and constructive remarks. We have taken the comments on board to improve and clarify the manuscript. Please find below a detailed point-by-point response to all comments.

Review Comments to the Author

Reviewer #1: The authors improved their manuscript “Does papillary muscle free strain has predictive value in risk stratification of patients with hypertrophic cardiomyopathy? But still some important points have to be mentioned:

LV outflow tract gradient measurements were only done at rest. The ESC risk stratification model includes gradients at provocations. As 50% of patients with obstructive HCM have only latent obstruction the authors underestimated risk scores in a significant number of patients without gradient measurement at provocation.

- We thank the reviewer. Measurements of LV outflow gradient were done in resting conditions. Patients who did not have resting LV outflow gradient underwent Valsalva maneuver in order to elicit latent obstruction. None of the patients in non-obstructive HCMP group had stress pressure gradients more than 30 mmHg. Patients who had labile (<30 mmHg at rest and ≥ 30 mmHg with Valsalva maneuver or other provocative tests) were not included in the study (we tried to involve patients with obstructive and non-obstructive type of HCMP). It has been explained in the material and methods section.

At page 5, line 21 the authors described “syncope” in general as risk factor. This is not correct as only “unexplained syncope” is an established risk factor for SCD and included in the risk stratification model. How many patients had unexplained syncope? If there are any – the statistics has to be done again.

- We thank the reviewer. Since only unexplained syncope is an established risk factor for sudden cardiac death in HCMP, we only included patients with a history of unexplained syncope in our study. Fifteen (27.2%) patients with HCMP had history of unexplained syncope in our study.

Reference 5 describes only a weak correlation between longitudinal strain and prediction for ICD.

- We thank the reviewer. Instead of reference 5, we used another meta-analysis which analyzed prognostic value of global longitudinal strain in hypertrophic cardiomyopathy and found that impaired left ventricular global longitudinal strain was associated with poor prognosis in HCMP patients.

Page8, lines 22/23: The reported correlations are weak.

- We thank the reviewer. In the present study we found moderate correlations of global longitudinal strain and anterolateral papillary muscle free strain with SCD risk score (r=0.480, p<0.001 and r=0.462, p<0.001, respectively). Our findings were in line with the previous data which found moderate correlation of strain values with maximal wall thickness, sudden cardiac death risk markers and other clinical and echocardiographic parameters in hypertrophic cardiomyopathy patients (1-4).

(1). Vergé MP, Cochet H, Reynaud A. Characterization of hypertrophic cardiomyopathy according to global, regional, and multi-layer longitudinal strain analysis, and prediction of sudden cardiac death. Int J Cardiovasc Imag. 2018;34(7):1091–1098.

(2). Rakesh K, Rajesh GN, Vellani H. 3D speckle tracking echocardiographic strain pattern in Hypertrophic Cardiomyopathy and its relation with Sudden Cardiac Death risk markers [Indian Heart J.](https://www.ncbi.nlm.nih.gov/pmc/articles/PMC8424279/) 2021 Jul-Aug; 73(4): 451–457. Published online 2020 Nov 20. doi: [10.1016/j.ihj.2020.11.144](https://doi.org/10.1016%2Fj.ihj.2020.11.144)

(3). Abozguia K, Nallur-Shivu G, Phan TT, Ahmed I, Kalra R, Weaver RA, et al. Left ventricular strain and untwist in hypertrophic cardiomyopathy: Relation to exercise capacity [Am Heart J.](https://www.ncbi.nlm.nih.gov/pmc/articles/PMC2877779/) 2010 May; 159(5): 825–832. doi: [10.1016/j.ahj.2010.02.002](https://doi.org/10.1016%2Fj.ahj.2010.02.002)

(4). Zhang L, Wan Y, He B, Wand L, Zhu D, Gao F. Left ventricular strain patterns and their relationships with cardiac biomarkers in hypertrophic cardiomyopathy patients with preserved left ventricular ejection fraction Front Cardiovasc Med . 2022 Oct 4;9:963110. doi: 10.3389/fcvm.2022.963110. eCollection 2022.

Page 9, line 4: 00.643-0.930

- We thank the reviewer. It has been corrected.

Page 9: Headline Discussion is missing

- We thank the reviewer. Headline Discussion has been added to the manuscript.

Page 10, lines 10-12: Reference 13 reports only weak correlations. This should be discussed.

- We thank the reviewer. Almaas et al. compared strain imaging with magnetic resonance imaging in detection of fibrosis in HCMP patient. They studied myectomy specimens in HCMP patients who underwent septal myectomy and histological fibrosis was classified as interstitial, replacement and total. Although they found moderate correlation of septal longitudinal strain with total and interstitial fibrosis, they did not find any correlation between late gadolinium enhancement on cardiac MRI and septal, interstitial or replacement. In that study, reduced longitudinal septal strain predicted ventricular arrhythmias in HCMP patients. According to the results of that study, compared to cardiac magnetic resonance imaging, functional assessment of myocardial functions by global longitudinal strain imaging might be a more valuable method in detecting cardiac fibrotic changes

Page 11, line 3: Reference 8 has no own data to support the statement.

- We thank the reviewer. Reference 8 has been deleted in that section.

Page 11, line 19: Reference 25 is no original paper, but an editorial which worked out that LGE >15% maybe an additional predictor for adverse outcome in HCM. This should be explained.

- We thank the reviewer. References regarding the prognostic value of strain imaging and late gadolinium enhancement in cardiac MRI studies have been added in the discussion section.

Legend of Figure 2 is wrong.

- We thank the reviewer. The order and the legends of the figures have been revised.

Figure 5 is not necessary.

- We thank the reviewer. Figure 5 has been removed.

Best regards.
